# Biases in COVID-19 Case and Death Definitions: Potential Causes and Consequences

**DOI:** 10.1017/dmp.2022.281

**Published:** 2022-12-12

**Authors:** Ronald B. Brown

**Affiliations:** School of Public Health Sciences, University of Waterloo, Waterloo, ON, Canada

**Keywords:** COVID-19, death definition, diagnostic case definition, genomic sequencing, information bias, media bias, nomenclature error, surveillance case definition

## Abstract

This paper investigates three controversies involving potential causes and consequences of information bias in case and death definitions during the coronavirus disease (COVID-19) pandemic. First, evidence suggests China’s surveillance data were biased and misinterpreted by the World Health Organization (WHO), prompting the WHO to advise nations to copy China’s lockdowns. China appeared to use narrow diagnostic definitions that undercounted cases and deaths. Second, novel genomic data disseminated during the pandemic without adequate guidance from rigorous epidemiologic studies biased infection control policies in many countries. A novel genomic sequence of a virus is insufficient to declare new cases of a novel disease. Third, media reports of COVID-19 surveillance data in many nations appeared to be biased. Broadened surveillance definitions captured additional information, but unadjusted surveillance data disseminated to the public are not true cases and deaths. Recommendations include clarification of the proper use of diagnostic and surveillance case and death definitions to avoid information bias.

Information bias is systematic error during the collection, analysis, interpretation, and publication of data, leading to false conclusions about the relationship between exposures and diseases.^1^ The present policy analysis investigates potential causes and consequences of information bias in coronavirus disease (COVID-19) case and death definitions, focusing on three controversies. The first controversy involves the World Health Organization’s (WHO) potential misinterpretation of China’s underreported surveillance case and death data, which may have misled the WHO into prompting nations to copy China’s lockdowns. The second controversy examines the activation of measures by nations to contain COVID-19 based on novel genomic sequencing data, without interpreting data from rigorous epidemiologic studies. The final controversy implicates media dissemination of unadjusted surveillance data that do not properly contextualize true COVID-19 cases and deaths. Errors in infection and disease nomenclature that occurred during the pandemic are also reviewed.

Grounded theory was used as a method to rigorously review the evidence in this paper.^2^ Findings were searched and selected from relevant public health documentation using Google, Google Scholar, PubMed, and other sources from the University of Waterloo Library. Current periodicals and credible international news media also provided information as pandemic events occurred in real time. Reviewed evidence was formed into themes and synthesized into a coherent narrative grounded in evidence.

## CHINA’S Novel Coronavirus Case Definitions

Within two years, from April 17, 2020, through April 16, 2022, China reported only six COVID-19 deaths and approximately 96 000 cases.^3^ During the same period, approximately 81.6 million US novel coronavirus cases with approximately 979 000 COVID-19 deaths were reported.^4^ Furthermore, after a 76-day lockdown was lifted in Wuhan, China, on April 8, 2020,^5^ China remained free from lockdowns for the next year, with the exception of two cities near Beijing in January, 2021.^6^ The WHO defines lockdowns as “large scale physical distancing measures and movement restrictions” that “can slow COVID-19 transmission by limiting contact between people."^7^ However, evidence suggests that China’s exceedingly narrow case definitions played a significant role in reducing China’s case and mortality statistics.

“A case definition is a set of standard criteria for classifying whether a person has a particular disease, syndrome, or other health condition.”^8^ Moreover, “using an inaccurate case definition may lead to misclassification bias,”^9^ a form of information bias. The WHO explained that case definitions should balance specificity and sensitivity for disease surveillance purposes.^10^ That is, overly narrow case definitions with inaccurate specificity exclude true cases, and overly broad case definitions with inaccurate sensitivity include false cases. The WHO emphasized that “a surveillance case definition is not intended to be used for diagnostic purposes, or for treating influenza or influenza-like illnesses.”^10^ Nevertheless, China reported COVID-19 surveillance cases and deaths that appeared to use diagnostic definitions.

Citing China’s 80% decline in cases during February 2020, a WHO-China Joint Mission on COVID-19 advised nations to do what China has done to “save lives and prevent thousands of cases of what is a very difficult disease.”^11^ Nations complied and copied China’s “unprecedented” lockdown measures to contain the virus.^12^ By February 22, 2020, China’s National Health Commission adopted the term *novel coronavirus pneumonia (NCP)* as China’s official disease name during the pandemic.^13^ To avoid an association with the previous SARS pandemic, China named the new virus, *2019-novel Coronavirus (2019-nCoV)*.^14^ Moreover, the National Health Commission’s policy tested for the virus exclusively in pneumonia patients,^15^ passing over less severe and asymptomatic respiratory infections and lowering case numbers. Additionally, patients without pneumonia were potentially less likely to be tested and confirmed as novel coronavirus cases, even if patients had a severe acute respiratory illness or condition known to be associated with SARS-CoV-2 and COVID-19, including respiratory syncytial virus,^16^ tuberculosis,^17^ bronchiolitis,^18^ and patients with mucociliary dysfunction—a condition that affects smell and taste.^19^

China’s National Health Commission listed NCP case definitions—“Moderate cases showing fever and respiratory symptoms with radiological findings of pneumonia”—including differential diagnosis criteria, in which “COVID-19 is mainly distinguished from other known viral pneumonia and mycoplasma [bacterial] pneumoniae infections.”^20^ Differential diagnosis further narrowed China’s case definitions by including only COVID-19 patients without coinfections. Of relevance, 1 study found that as many as 86% of patients infected with SARS-CoV-2 in March and April 2020 were coinfected with additional respiratory pathogens,^21^ but that this high rate may be due to selective testing of more severe cases in early 2020. A more recent worldwide meta-analysis found 20.97% bacterial, 12.58% viral, and 12.60% fungal coinfections in COVID-19 patients.^22^ Failure to recognize information bias in China’s exceedingly narrow diagnostic case definitions may have misled the WHO into prompting other nations to reduce cases and deaths by following China’s lockdowns. Eventually, the WHO admitted that lockdowns were not having the same level of success in other countries, and, at a May 13, 2020, briefing, WHO officials reluctantly declared, “This virus may never go away.”^23^ Recently, the WHO’s Director-General claimed that China’s zero-COVID policy is not “sustainable.”^24^ With more widespread testing now compared to February 2020, it is much more apparent that lockdowns aren’t containing the spread of COVID-19.

## Genomic Epidemiology

Over approximately the last 15 years, the identification of novel viral species accelerated with the development of genome-sequencing technology.^25^ However, a newly discovered genomic sequence of a virus is insufficient to declare new cases of a novel disease without more rigorous human studies.^26^ Epidemiology is not keeping pace with advances in genomic sequencing technologies to “bridge the gap between genomic epidemiology and real-life infection control.”^27^ Large cohort studies are needed to evaluate the association of emerging viruses and variants with epidemiological factors such as demographic characteristics, socioeconomic data, and predisposing comorbidities.^28^

Based on a newly discovered genomic sequence, the WHO notified nations in February 2020 that the unknown virus of concern “is not SARS and this is not influenza.”^11^ Lacking data from epidemiologic studies for guidance, the WHO warned countries to “activate and scale up your emergency response mechanisms” and “take urgent and aggressive action.”^29^ In response, Stanford University epidemiologist John Ioannidis warned that “we are making decisions without reliable data.”^30^

In the meantime, the preliminary evaluation expert group that discovered the genomic sequence of the novel coronavirus from Wuhan acknowledged that further steps were needed “to combine etiological research, epidemiological investigation and clinical manifestations for expert research and judgment.”^31^ Although Wuhan cases were initially identified as viral pneumonia of unknown etiology,^32^ cases may not have been as novel as implied by China’s dysfunctional surveillance system for detecting pneumonia of unknown etiology (PUE). A 2016 survey from Chinese and US Centers for Disease Control and Prevention (CDC) showed that China’s PUE system used a very broad case definition that could lead to “hundreds of thousands” of cases a year.^33^ The survey noted that national PUE training had been lacking in China since 2008, and “most clinicians have limited awareness of and are not reporting to the PUE system,” suggesting that many cases similar to the Wuhan cases remained unreported.

## WHO Surveillance Case and Death Definitions

A common error in the medical subspecialty of infectious diseases assumes that “infection is synonymous with disease.”^34^ But infection alone, the multiplication of microbes within the body, may be insufficient to lead to disease without signs and symptoms of illness or cell damage from the infection.^35^ The WHO appeared to commit a nomenclature error by naming the pandemic disease *novel coronavirus disease 2019 (COVID-19)*, which is synonymous with the WHO’s original name for the virus, *2019-novel coronavirus (2019-nCoV)*.^36^ The WHO’s initial avoidance of the virus name *SARS-CoV-2* appears to have been motivated by the desire to disassociate the pandemic from the SARS 2003 pandemic, which burdened China’s economy.^37^

The WHO defined a confirmed case in March 2020 as “a person with laboratory confirmation of COVID-19 infection, irrespective of clinical signs and symptoms.”^38^ Medical experts objected to the absence of disease or injury in the WHO’s surveillance definitions,^39^ and the conflation of the viral infection with the disease was considered “an unforgiveable breach of a first rule in infectiology.”^40^ Such confusion may arise if public health authorities fail to convey to the public that the use of surveillance case definitions is intended to collect data across the full range of cases and infections (severe, moderate, mild, and asymptomatic) and is not intended for diagnostic purposes.

The WHO defined a COVID-19 death in August 2020 as “a death resulting from a clinically compatible illness in a probable or confirmed COVID-19 case, unless there is a clear alternative cause of death that cannot be related to COVID-19 disease (e.g. trauma).”^41^ In contrast with China’s narrow definitions, the WHO’s definition increased surveillance sensitivity to include many more deaths beyond the boundaries of severe acute respiratory illness, as evidenced by the CDC’s report listing unrelated conditions contributing to US death certificates for COVID-19.^42^

Tsang et al. reported that broadened COVID-19 case definitions helped gather additional information about the extent of the infection, “particularly milder cases and those without epidemiological links.”^43^ The researchers advised that broadened case definitions should be adjusted “to avoid bias” when estimating the true epidemic growth rate and infection rate. Nevertheless, many nations failed to adjust data accordingly, and biased COVID-19 cases and death rates were reported in the media that did not properly “contextualize” true cases and deaths.^44,45^

## Summary

**Figure 1.**
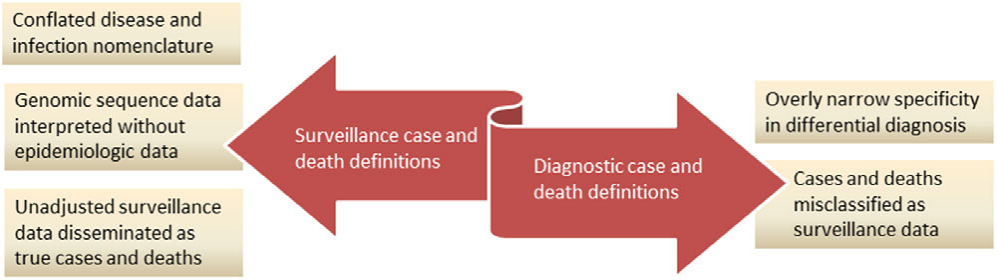
Bias in COVID-19 case and death definitions.

Figure 1 summarizes information bias in COVID-19 case and death definitions discussed in this paper. China appeared to use narrow diagnostic case definitions to underreport novel coronavirus cases and deaths as surveillance data, which prompted the WHO to recommend that other nations adopt China’s unprecedented lockdowns. Public health responses to contain the novel coronavirus and variants were based on genomic surveillance data without sufficient guidance from data of epidemiologic studies. The WHO’s surveillance case definitions conflated disease and infection nomenclature by assigning the disease a name synonymous with the virus, and laboratory-confirmed infections were sufficient to confirm disease cases. In contrast with China’s narrow diagnostic data, broad surveillance data disseminated by the media in other nations were not adjusted to represent true cases and deaths. The present paper recommends greater clarification in the proper use of diagnostic and surveillance case and death definitions to avoid information bias. More studies are needed to examine additional evidence and quantitative analyses of COVID-19 cases and deaths in nations across the globe.
